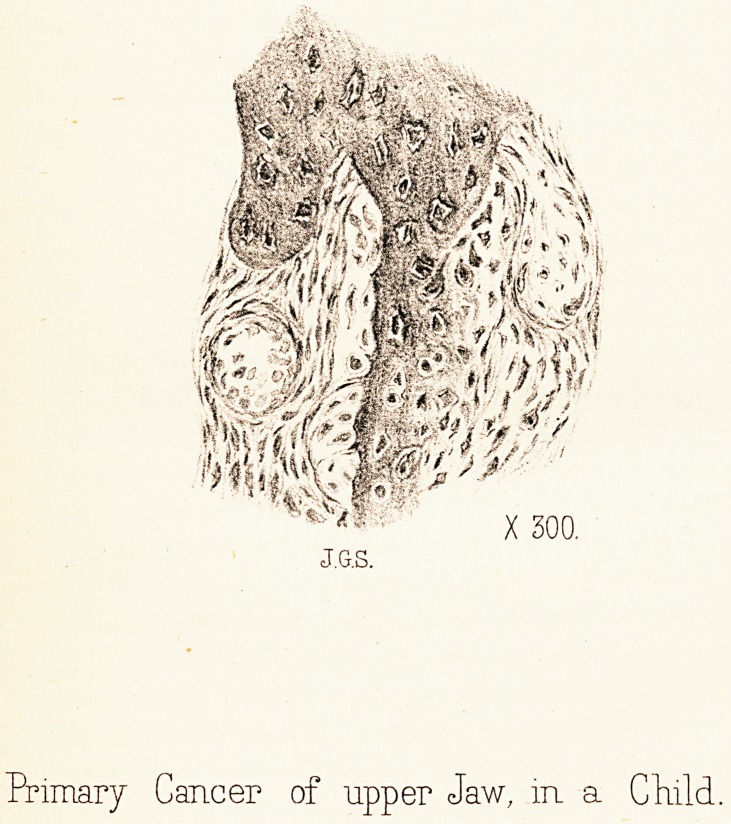# Primary Scirrhus of the Upper Jaw in a Child Two Years Old

**Published:** 1883-07

**Authors:** 


### PRIMARY SCIRRHUS OF THE UPPER JAW IN A CHILD TWO YEARS OLD.

Hard cancer as a primary disease of the jaws is almost unknown. Mr. Pollock, in the new edition of *Holmes's System of Surgery*, writes:—"Scirrhus in its

Plate IX.

X 300.

J.G.S.

Primary Cancer of upper Jaw, in a Child.

onslaughts appears to disregard these regions; for though found as a secondary condition in bone, it has not fallen to our lot to notice it in the bones of the face: the experience of others confirms this observation." Heath, however, says in his work on *Diseases of the Jaws* that, though he has met with no specimen himself, he believes preparation 1059 E in the College of Surgeons' Museum, removed by Coates of Salisbury, to be one of scirrhus of the upper jaw.

On January 30th, 1883, Mr. Prichard, at the Bristol Royal Infirmary, removed from a girl two years old the right upper jaw for what at the time was believed to be medullary cancer. The tumour depressed the hard palate, pushed aside the nasal bones, and raised the floor of the orbit. The skin at the naso-orbital angle was red and discoloured, as if ulceration were about to take place. It had been growing for so long a period as eighteen months. The wound healed by first intention, and the child was dismissed in a fortnight. It had recurred when the child was last seen.

Mr. Prichard kindly gave me the tumour to examine, and I believe it to be an undoubted case of primary scirrhus. Everywhere the appearance of the developed growth was that of hard cancer, and beautiful examples of the manner in which this growth invades bone were met with. A sketch of one of the portions is given opposite (Pl. IX.).

## Figures and Tables

**Figure f1:**